# Retraction: What is the optimal In2Care stations density to achieve *Aedes aegypti* population reduction in a dengue endemic setting?

**DOI:** 10.1371/journal.pntd.0014866

**Published:** 2026-11-12

**Authors:** The PLOS Neglected Tropical Diseases Editors

After this article [1] was published, the following concerns about the results and statistical analyses were raised:

1. None of the three underlying dataset versions available through Figshare [2] appear to match all results presented in the article [1].
2. For each dataset version available in [2], when all study site results are combined, the maximum number of eggs in the “After” period appears to be exactly 201 eggs, with multiple trap-week results showing 201 eggs, such that the dispersion of data in the “After” period appears to show an unexpected maximum cut-off value. This maximum egg number is not observed in the “Before” or “During” periods.
3. Some of the OPI and ODI results in Table 2 do not appear to match the underlying datasets presented in [2].
4. Fig 3 displays egg counts up to 100 on the y-axis, although some underlying trap-week counts exceed 100 eggs. It is unclear whether those higher counts were included when calculating the plotted Ovitrap Density Index (ODI) summaries.
5. The Funding statement in [1] states the authors received no specific funding for the work, despite thousands of In2Care stations being used in [1].

Regarding point 1 above, the corresponding author clarified that version 1 of the dataset shows all ovitraps handled by the municipality of Goiânia during the 62 weeks of study in the selected neighborhoods. Some of the traps were already being monitored by Goiânia health agents before the commencement of the study in [1], and more ovitraps were added to each site of interest in the first week of intervention. The corresponding author stated that versions 2 and 3 of the dataset in [2] are the same, and that these versions show reorganization of the dataset to ensure consistence with the revised analytical framework and hypothesis in [1]. As the new analytical framework shown in [1] involved comparing egg counting only within each area, the same number of traps before, during, and after interventions was needed, and the ovitraps added only during the intervention phase were removed in versions 2 and 3 compared to version 1 of the dataset. The corresponding author stated that version 1 of the dataset was used to prepare Table 1, Fig 2, Fig 4, and version 3 of the dataset as used to prepare the GLMM analysis and Table 2. It remains unclear which dataset version was used to prepare Fig 3 in [1]. The *PLOS Neglected Tropical Diseases* editors remain concerned that all results, tables and figures in [1] are not generated using the same dataset, meaning the results may not be directly comparable.

Regarding point 2 above, the corresponding author clarified that the original datasets were not originally generated for research purposes and instead derive from a nationwide surveillance initiative led by the Brazilian Ministry of Health initiated in 2022. The corresponding author provided details about how the ovitraps were processed and counted, and stated no instructions were issued to establish an upper threshold for the number of eggs to be recorded on an individual ovitrap panel. The *PLOS Neglected Tropical Diseases* Editors remain concerned that the validity and reliability of the “After” period data cannot be verified.

The corresponding author agreed with the concerns in point 3 above. They stated the results in Table 2 do not appear to match the underlying datasets, the denominator used in the ODI results in Table 2 was incorrect, and they provided a replacement Table 2. Upon editorial review, the PLOS Neglected Tropical Diseases Editors noted that some of the updated results in Table 2 do not appear to support the results and conclusions of the published Table 2 in [1]. In addition, PLOS remains concerned that the ODI calculation errors in Table 2 in [1] may affect the ODI results presented in Figs 3 and 4.

Regarding point 4 above, an external expert noted that if egg counts above 100 were excluded before calculating the weekly ODI summaries, those summaries would be affected, whereas restricting only the displayed y-axis range would leave the calculated summaries unchanged. The external expert stated clarification on whether all egg counts were included and how Fig 3 had been generated was required.

The corresponding author provided a replacement version of Fig 3 that was plotted using all data and not only the 0–100 interval data. Upon review, the *PLOS Neglected Tropical Diseases* Editors noted that some of the ODI and confidence interval results appear different to the published results in [1].

Regarding the funding of [1], the corresponding author clarified the In2Care units were procured by the municipal health department of Goiânia as the study in [1] is part of a public health intervention. Authors WT, MR, and PGGM are employed by the Municipality of Goiânia. With this information, the *PLOS Neglected Tropical Diseases* Editors consider point 5 above resolved.

In light of the above unresolved concerns that question the reliability of the reported results, the *PLOS Neglected Tropical Diseases* Editors retract this article.

RMDF did not respond to the final editorial decision. WT, MR, PGGM, LHPV, ASL, and WACDC either did not respond directly or could not be reached.

In addition to the points above, concerns were raised about the generalized linear mixed model (GLMM) statistical analysis approach, including whether the use of a nested random effects structure was appropriate, convergence errors generated in R when using a nested random effects structure, and very large absolute Z ratios reported in the High-density results in Table 3. The corresponding author provided model diagnostics and reanalyses of their data; however, due to the unresolved concerns described above which supported retraction of this article, PLOS did not investigate the GLMM concerns further.
